# Evolutionary dynamics of organised crime and terrorist networks

**DOI:** 10.1038/s41598-019-46141-8

**Published:** 2019-07-05

**Authors:** Luis A. Martinez-Vaquero, Valerio Dolci, Vito Trianni

**Affiliations:** 1 Institute of Cognitive Sciences and Technologies, National Research Council of Italy, via San Martino della Battaglia 44, 00185 Rome, Italy; 2 Present Address: Lab of Socioecology and Social Evolution, Department of Biology, KU Leuven, Naamsestraat 59, 3000 Leuven, Belgium; 3 INFN Roma1, Rome, Italy; 4 Physics Department, Sapienza University of Rome, Rome, Italy

**Keywords:** Statistical physics, thermodynamics and nonlinear dynamics, Mathematics and computing

## Abstract

Crime is pervasive into modern societies, although with different levels of diffusion across regions. Its dynamics are dependent on various socio-economic factors that make the overall picture particularly complex. While several theories have been proposed to account for the establishment of criminal behaviour, from a modelling perspective organised crime and terrorist networks received much less attention. In particular, the dynamics of recruitment into such organisations deserve specific considerations, as recruitment is the mechanism that makes crime and terror proliferate. We propose a framework able to model such processes in both organised crime and terrorist networks from an evolutionary game theoretical perspective. By means of a stylised model, we are able to study a variety of different circumstances and factors influencing the growth or decline of criminal organisations and terrorist networks, and observe the convoluted interplay between agents that decide to get associated to illicit groups, criminals that prefer to act on their own, and the rest of the civil society.

## Introduction

Criminal organisations (COs) and terrorist networks (TNs) represent different outcomes of a similar process, whereby individuals join together into illicit groups to bring forth criminal activities at the expenses of the civil society that they want to exploit or intimidate^1,2^. Despite being inspired by different objectives and modus operandi, theories have been advanced about organised crime and terrorism laying at the two extremes of a continuum, in which differences get blurred in a mix of illegal and violent activities aimed at gaining both economical power and supremacy over the states^3,4^. It is not uncommon to see criminal organisations engaged in terror tactics, or terrorist networks perpetrating all sorts of criminal activities^5–8^. Although empirical evidence about the relevance of a deep crime-terror nexus is still scarce, it is reasonable to consider that the growth or decline of COs or TNs are bound to similar dynamics that pertain their sustainability and attractiveness towards possible recruits.

Modelling crime through the lenses of physics^9^ or through computational approaches^10^ can provide useful insights to understand the dynamics and forces underlying its development in relation to law enforcement strategies, and to evaluate rehabilitation programs^11–15^. Different approaches have been attempted to model criminal behaviour, from abstract dynamical systems models^16^—possibly including spatial factors influencing crime diffusion^17,18^—to models following an evolutionary game theory approach, in which players display competitive strategies (e.g., criminals versus punishers) and interact within the context of an adversarial game^11,19–22^. However, despite a raising interest into crime dynamics from a complex systems perspective, organised crime and terrorism received relatively little attention to date^23,24^. Agent-based models have been proposed in the context of extortion racket^25,26^. A similar approach has been taken to study fundamentalism and radicalisation in the context of terrorism^27–30^, but here attention has been directed mainly towards the analysis of network properties and the evaluation of disruption interventions^28,31–35^. Recruitment to organised crime and terrorism has not been thoroughly investigated so far, despite being at the core of the dynamics of such groups, and should therefore be one basic ingredient for any modelling study that aims at unveiling the complex interactions underlying CO/TN dynamics.

In this study, we introduce a stylised model that can be easily parametrised to represent both the dynamics of COs and of TNs. Similarly to previous studies^11,19^, we introduce a *N*-person adversarial game that contrasts agents engaging in illicit activities and regular *honest* individuals that instead may just lose out. Differently from previous studies, we differentiate the criminal population into agents associated into COs or TNs, and individual agents acting on their own—be they criminals not structured within a CO, or terrorists not belonging to a TN, hereafter both referred to as *lone wolves* for brevity. In this way, we can identify the conditions under which joining a criminal or terrorist organisation is advantageous. We consider different possible interactions among the players, taking into account the effects of punishment from institutions, from the CO/TN itself as well as social control enacted by the civil society^36^. Punishment represents a strong driver for positive behaviour, as several theoretical and experimental studies demonstrated^37–40^. Indeed, punishment is at the basis of law enforcement strategies, and also permeates the world of COs and TNs, making it a fundamental mechanism to consider for the study of CO/TN dynamics^19,21^. When focusing on COs, we observe that under certain conditions criminals contribute to the eradication of non-organised crime by lone wolves, actually providing a form of protection to the civil society, as suggested by criminological theories^41,42^. When focusing on TNs, instead, we identify conditions from which TNs coexist in an equilibrium with lone wolves, effectively benefiting from their illicit activities without paying related costs^43,44^. In the following, we will describe the details of the proposed stylised model, and we discuss the dynamics characterising the above mentioned cases, and beyond.

## Methods

We consider an adversarial game in a multi-agent framework, in which agents play in small groups formed randomly from a larger well-mixed population. The latter represents a given community—within a quartier, a city or a region—that is tight enough to make well-mixed interactions possible. The small groups within which adversarial games are played represent instead temporary gatherings that may form and disband at any time. Agents within the population can take one of the following roles: (i) *honest citizens H* not committing any illicit action; (ii) *criminals C* associated to a CO or a TN, who share the benefits and burden of their actions; and (iii) *lone wolves W* acting independently from any organisation. The game is divided into two phases: the *acting stage*, in which some criminal activity may take place by a *victimiser* (wolf or criminal) to the detriment of a subset of agents *victims* within the group; and the *investigation stage*, in which victimisers may get punished, either from some state institutions or from other individuals within the group. Note that we use the term *crime* for both criminal and terrorist actions. After a certain number of rounds, the evolutionary dynamics take place on the basis of the payoff cumulated by every agent, modelling the opportunistic change of strategies within the population. We study this adversarial game with an analytical mean field model and complement it with Monte Carlo simulations, as detailed below.

### Mean field evolutionary model

We first discuss the details of the proposed adversarial game in the context of a mean field approximation with replicator dynamics^45^, where we calculate the average payoff that each agent obtains after an infinite number of rounds played in every possible configuration of groups in the population.

We consider a well-mixed population of *Z* individuals. Within the overall population, each role *k* is present with a fraction *x*_*k*_ = *Z*_*k*_/*Z*, where $\sum {Z}_{k}=Z$ and *k* = {*H*, *C*, *W*} stands for honest, criminal and lone wolf, respectively. We will use the notation 〈*Z*_*H*_, *Z*_*C*_, *Z*_*W*_〉 to indicate a population formed by *Z*_*H*_ honest citizens, *Z*_*C*_ criminals, and *Z*_*W*_ lone wolves. From such population, individuals are randomly chosen to form groups of *N* individuals, and each group is composed by *N*_*k*_ individuals of each type. Similarly, we refer to a specific group configuration as 〈*N*_*H*_, *N*_*C*_, *N*_*W*_〉.

#### Acting stage

After a group is set up, one agent is taken randomly from the group to perform its predefined action. The probability that any given player is chosen from the group is then *p*_1_ = 1/*N*, while the probability of choosing a player with role *k* is *p*_*k*_ = *N*_*k*_/*N*. If a honest agent is selected (with probability *p*_*H*_), nothing happens. Instead, when the selected agent is a criminal (with probability *p*_*C*_) or a lone wolf (with probability *p*_*W*_), some criminal action is performed. In the case of lone wolves, the probability of acting is reduced by a factor ${p^{\prime} }_{W}=1-\delta (1-{p}_{C})$, which represents the correlation between the presence of criminals within the group and the likelihood that lone wolves take action. For instance, in the terrorism scenario, lone wolves would act mainly when driven by propaganda from TNs (*δ* = 1), and would otherwise stay quiescent.

Whenever the chosen player is a criminal or a wolf, she will cause a damage with value *c*_*k*_ in each one of the victims in the group and obtain a benefit *r*_*k*_*c*_*k*_ from each one of them. If the victimiser is a criminal, honests and wolves are the only victims and the obtained benefits are shared among all the criminals within the group (since criminals belong to the same organisation, they act as a group: they do not damage each other but rather share the benefits of their actions). The average benefit *b*_*C*_ obtained by criminals and the average damage *d*_*C*_ caused on others can be computed as follows:

1 $${b}_{C}=\frac{1}{{N}_{C}}{r}_{C}{c}_{C}{p}_{C}(N-{N}_{C})={r}_{C}{c}_{C}(1-{p}_{C}),\,{d}_{C}={c}_{C}{p}_{C}.$$

On the other hand, if this victimiser is a wolf, her victims are all the other members of the group, including other wolves and criminals. Hence, the average benefit *b*_*W*_ and the average damage *d*_*W*_ can be computed as follows:

2 $${b}_{W}={r}_{W}{c}_{W}(N-1){p}_{1}{p^{\prime} }_{W},\,{d}_{W|{N^{\prime} }_{W}}={c}_{W}{p^{\prime} }_{W}\frac{{N^{\prime} }_{W}}{N},$$

where ${N^{\prime} }_{W}$ represents the number of wolves in the group other than the focal player, and is specified to take into account the probability that a wolf different from the focal player commits a crime. Specifically, ${N^{\prime} }_{W}={N}_{W}$ when considering the damage inflicted by wolves to honests and criminals, while ${N^{\prime} }_{W}={N}_{W}-1$ when considering the damage inflicted by a wolf on the other wolves in the group. Finally, we consider the possibility that a fraction *τ* of the benefit obtained by wolves is actually benefiting the criminals. For instance, a terrorist network would gain in reputation and power also when the criminal activity is executed by lone wolves without paying any cost for it.

Overall, from Eqs (1) and (2), it is possible to compute the average payoffs in the acting stage ${w}_{k}^{A}$, as follows:

3 $${w}_{H|\langle {N}_{H},{N}_{C},{N}_{W}\rangle }^{A}=-\,{d}_{C}-{d}_{W|{N}_{W}}$$

4 $${w}_{C|\langle {N}_{H},{N}_{C},{N}_{W}\rangle }^{A}={b}_{C}+\tau {b}_{W}-{d}_{W|{N}_{W}}$$

5 $${w}_{W|\langle {N}_{H},{N}_{C},{N}_{W}\rangle }^{A}=(1-\tau ){b}_{W}-{d}_{C}-{d}_{W|{N}_{W}-1}$$

It is possible to notice that honests are only harmed by others in this stage, while criminals and wolves can get a benefit from illicit actions, but also suffer from the criminal activities of other individuals within the group.

#### Investigation stage

After each criminal act, an investigation is conducted. To this end, individuals are chosen from the group and a control is made on them to ascertain if they committed a crime. If the victimiser is found, she will receive a punishment. We consider three types of investigations and corresponding punishments:

- **State**: an investigation performed by a law enforcement organisation against any victimiser. The law enforcement organisation is not modelled explicitly in the multi-agent framework, but as a super-agent. The effects of the corresponding investigation are included through the parameter *β*_*S*_, which represents the level of punishment inflicted to any victimiser and is independent from the group/population configuration.
- **Civil**: social control carried out by the civil society—i.e., honest individuals—against any victimiser. When successful, this type of investigation leads to the punishment *β*_*H*_ to be inflicted to the victimisers. In this case, the probability of success of such an investigation is proportional to the fraction of honest individuals *p*_*H*_.
- **Criminal**: an investigation performed by the criminal organisation against its potential rivals, the wolves. Punishment is controlled by the parameter *β*_*C*_ and the probability of success for the investigation is proportional to the fraction of criminals *p*_*C*_.

Note that wolves can receive a higher level of punishment since also criminals may punish them. In the case of criminals, instead, we consider that being part of a CO can lead to the punishment of any members within the group. The investigated criminal will receive the full punishment and her partners will receive that punishment reduced by a factor *γ*, since capturing a criminal can lead to capturing the rest of the criminals involved in the organisation. Overall, criminals are easier to identify than wolves due to chance only, but the former have the capacity to punish the latter. The reductions in the payoffs that each type of victimiser is obtaining from this stage ${w}_{k}^{I}$ are, in average, as follows:

6 $${w}_{H|\langle {N}_{H},{N}_{C},{N}_{W}\rangle }^{I}=0$$

7 $${w}_{C|\langle {N}_{H},{N}_{C},{N}_{W}\rangle }^{I}=-\,({\beta }_{S}+{\beta }_{H}{p}_{H}){p}_{C}[\gamma {p}_{C}+(1-\gamma ){p}_{1}]$$

8 $${w}_{W|\langle {N}_{H},{N}_{C},{N}_{W}\rangle }^{I}=-\,({\beta }_{S}+{\beta }_{H}{p}_{H}+{\beta }_{C}{p}_{C}){p}_{1}^{2}{p^{\prime} }_{W}$$

In computing these payoffs, we model the fact that an agent must first commit a criminal act, which happens with probability ${p}_{1}{p^{\prime} }_{W}$ for wolves and *p*_*C*_ for criminals, and then gets punished upon investigation, which happens with probability *p*_1_ for wolves and [*γp*_*C*_ + (1 − *γ*)*p*_1_] for criminals, to account for the collective punishment discussed above.

#### Evolutionary dynamics

In order to compute the average payoffs *ω*_*k*_ that each type of individual *k* is obtaining in a given population, we compute the average payoff that this individual is getting in all the possible groups she can be part of, considering all the combinations of remaining *N* − 1 individuals in the group. More specifically, the group of *N* individuals 〈*N*_*H*_, *N*_*C*_, *N*_*W*_〉 is formed by the focal player *k* and a subgroup ${\langle {N^{\prime} }_{H},{N^{\prime} }_{C},{N^{\prime} }_{W}\rangle }_{k}$ such as ${N}_{k}={N^{\prime} }_{k}+1$ and ${N}_{\tilde{k}}={N^{\prime} }_{\tilde{k}}$ for $\tilde{k}\ne k$. The subgroup is drawn from the population excluding the focal player ${\langle {Z^{\prime} }_{H},{Z^{\prime} }_{C},{Z^{\prime} }_{W}\rangle }_{k}$ with ${Z}_{k}={Z^{\prime} }_{k}+1$ and ${Z}_{\tilde{k}}={Z^{\prime} }_{\tilde{k}}$ for $\tilde{k}\ne k$. The likelihood of obtaining ${\langle {N^{\prime} }_{H},{N^{\prime} }_{C},{N^{\prime} }_{W}\rangle }_{k}$ drawn from ${\langle {Z^{\prime} }_{H},{Z^{\prime} }_{C},{Z^{\prime} }_{W}\rangle }_{k}$ is given by the multivariate hypergeometric distribution ${\mathcal H} \,[{\langle {N^{\prime} }_{H},{N^{\prime} }_{C},{N^{\prime} }_{W}\rangle }_{k}]$:

9 $${\mathscr{H}}[{\langle {N^{\prime} }_{H},{N^{\prime} }_{C},{N,}_{W}\rangle }_{k}]=\{\begin{array}{cc}\frac{(\begin{array}{c}{Z^{\prime} }_{H}\\ {N^{\prime} }_{H}\end{array})(\begin{array}{c}{Z^{\prime} }_{C}\\ {N^{\prime} }_{C}\end{array})(\begin{array}{c}{Z^{\prime} }_{W}\\ {N^{\prime} }_{W}\end{array})}{(\begin{array}{c}Z-1\\ N-1\end{array})} & {\rm{i}}{\rm{f}}\,{\rm{\forall }}j\,{N^{\prime} }_{j}\le {Z^{\prime} }_{j}\\ 0 & {\rm{o}}{\rm{t}}{\rm{h}}{\rm{e}}{\rm{r}}{\rm{w}}{\rm{i}}{\rm{s}}{\rm{e}}\end{array}$$

The average payoff can be then computed starting from Eqs 3–8, and weighting the payoff obtained in each subgroup with ${\mathcal H}$:

10 $${\omega }_{k}=\sum _{{N^{\prime} }_{C}=0}^{N-1}\,\sum _{{N^{\prime} }_{W}=0}^{N-{N^{\prime} }_{C}-1}\,{\mathscr{H}}[{\langle {N^{\prime} }_{H},{N^{\prime} }_{C},{N^{\prime} }_{W}\rangle }_{k}]({w}_{k|\langle {N}_{H},{N}_{C},{N}_{W}\rangle }^{A}+{w}_{k|\langle {N}_{H},{N}_{C},{N}_{W}\rangle }^{I}),$$

We assume that the dynamics of the system follow the replicator dynamics equation. For each subpopulation, we compute the direction and strength of change as follows:

11 $${\dot{x}}_{k}={x}_{k}({\omega }_{k}-\bar{\omega }),$$

where $\bar{\omega }={\sum }_{i}{x}_{i}{\omega }_{i}$ is the average payoff including every individual of the population. In order to calculate the most important (or most visited) configurations of the finite-size population, we assume that in each evolutionary time-step, only one individual can change state. From each possible configuration 〈*Z*_*H*_, *Z*_*C*_, *Z*_*W*_〉, we calculate the closer next point on the trajectory determined by the replicator dynamics from Eq. 11.

In this way, we can build the transition matrix among all the possible configurations of the population (note that to avoid numerical issues, we add a small probability of *μ* = 10^−6^ to every transition, and we renormalise all transition probabilities afterwards ensuring a correct transition matrix for the Markov chain). The stationary distribution from the Markov chain represented by this matrix in then computed. The probabilities in the stationary distribution represent the importance of each configuration or, in other words, the time that the system spends in each configuration point.

### Monte carlo simulations

We developed a multi-agent simulation with the purpose of validating the results from mean-field approximations as discussed above. Mean field approximations and the replicator dynamics are based on a good estimation of the average payoff, which determines the outcome of the evolutionary process. In real systems, estimations are noisy and bound to many factors, such as the group size or the particular settings of the studied game. It is therefore important to verify the validity of the analytical results in the light of the available knowledge.

Simulations are implemented following the same stages as discussed above. Also in this case, a population of *Z* individuals evolves with individuals changing their role between honest, organised criminals and lone wolves. To compute the payoff *ω*_*j*_ for each agent *j*, *G* games are played, and in each game the population *Z* is partitioned in *Z*/*N* groups that undergo the acting and investigation stages. Payoffs are assigned to each individual and cumulated across different games. After each game, the population is reshuffled and partitioned to generate another set of data. Once computed an average payoff for each individual over the *G* games, the evolutionary step takes place by selecting two players at random and having them change their role probabilistically according to their relative payoffs. The probability that agent *i* copies the role of agent *j* is computed according to the Fermi function:

12 $$P({k}_{i}\leftarrow {k}_{j})=\frac{1}{{e}^{-({\omega }_{j}-{\omega }_{i})/T}+1}$$

where *T* is a parameter determining the steepness of the sigmoid function. With a small probability *μ*, mutations take place instead, and one randomly selected individual chooses among the three roles with equal probability. Simulations have been optimised to efficiently compute the evolutionary dynamics over a long time, so as to identify the trajectory of the system.

## Results

The dynamics grasped by the proposed model are determined by the chosen parameterisation in a non-intuitive way. We exploit the possibilities offered by the model to represent different contexts by fixing a number of parameters that characterise the criminal scenario (e.g., the influence *δ* played by criminals on the acting probability of lone wolves), and for each context we study the importance of the different types of investigation and punishment onto the population dynamics by varying *β*_*S*_, *β*_*H*_, and *β*_*C*_. For each combination of these parameters, we study the dynamics and calculate the basins of attraction of the population dynamics by looking at the stationary distribution of the different individual types, therefore identifying when criminals prevail over lone wolves or vice versa, or when crime remains under control or gets totally eradicated from society.

### Organised crime

We start by exploring a scenario representing the presence of a CO within our abstract society. In this case, we consider that there is no favourable interaction between the CO and the lone wolves acting independently, neither in the probability of committing a crime (i.e., *δ* = 0) nor in the remission of any benefit (i.e., *τ* = 0). In practice, criminals and wolves are in competition: they commit crimes independently one from the other, harm each-other and keep the benefit resulting from their criminal action for themselves. Without loss of generality, we assume that wolves and criminals produce the same harm and obtain the same benefit from it, hence *c*_*W*_ = *c*_*C*_ and *r*_*W*_ = *r*_*C*_ = 1. When not stated otherwise, we assume that punishment to criminals other than the investigated one is halved (i.e., *γ* = 0.5).

**Figure 1:**
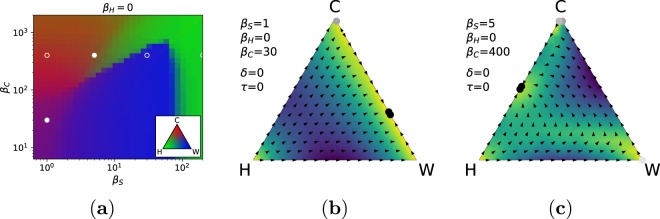
Effect of punishment from the state (*β*_*S*_) and criminal organisation (*β*_*C*_) when the civil society does not participate to the investigation phase (*β*_*H*_ = 0). (**a**) Proportion of honest individuals (green), lone wolves (blue) and criminals (red) in the stationary distribution for different values of *β*_*S*_ and *β*_*C*_. The inset shows the colour-coding corresponding to every point in the simplex representing the percentage of each individual type within the population. Circles correspond to representative configurations displayed in the accompanying panels (filled circles) or in the Supplementary Fig. S1 (empty circles). (**b**,**c**) Simplex describes the dynamics of the system for *β*_*C*_ = 400 and different values of *β*_*S*_. Arrows represent the direction of change for the three sub-populations, while the background colour represents the intensity of change—the darker the stronger. Filled circles indicate the most visited states: the grey scale represents the corresponding probability in the stationary distribution—the darker the higher—normalised according to the most visited state and using a minimum threshold of 10%. Parameters of the model for this figure: *γ* = 0.5, *N* = 10, *c*_*W*_ = *c*_*C*_ = *r*_*W*_ = *r*_*C*_ = 1, *Z* = 50.

We first consider the case in which the civil society is not contributing to investigations and punishment (i.e., *β*_*H*_ = 0). In Fig. 1, we represent the proportion of each individual type in the stationary distribution for a wide range of values for *β*_*S*_ and *β*_*C*_, as well as the system dynamics for representative combinations. The stationary distributions reveal the presence of different possible regimes, and indicate that punishments from the state organisation and from criminals interact in a non-trivial way (see Fig. 1a). As expected, when punishment from criminals is relatively small (*β*_*C*_ < 50), wolves take over the population, unless the punishment *β*_*S*_ is also very small. In correspondence of a weak state, there exist a region in which criminals and wolves coexist at the expenses of the civil society (see Fig. 1b), and also a region where the CO proliferates by increasing punishment against lone wolves (as shown in Fig. 1c, where *β*_*C*_ = 400). In this latter condition, the CO practically takes over the role of the state in punishing criminal acts carried out by wolves, providing a form of protection^41^. The model predicts that, in correspondence of a low punishment from the state organisation, criminals and honests can coexist in the society. With increasing levels of punishment from the state organisation, the CO gradually disappears to the benefit of the civil society. However, an undersized CO also has a low control potential against lone wolves, leaving room to their proliferation: when a specific value of *β*_*S*_ is reached (see for instance *β*_*C*_ = 400 and *β*_*S*_ = 200 in Fig. 1), a phase transition takes place and wolves take over the entire population. Indeed, *β*_*S*_ is high enough to undermine the power of criminals but not to punish efficiently wolves. Without criminals able to control wolves and a state not strong enough to do it by itself, the population is at mercy of wolves. Only with a sufficiently strong state punishment (e.g., *β*_*S*_ > 100) crime can get completely eradicated (see Fig. 1a and the Supplementary Fig. S1). The fact that wolves can have an advantage over criminals for medium values of *β*_*S*_ finds its explanation in the way in which investigations are modelled, which make the identification of a criminal group much more likely than a single lone wolf. This is a simplification introduced by the model, which however shows the interesting effects introduced by a differential probability of being caught. Additionally, punishment is not limited to the investigated criminal, but also affect the other criminals in the group via the factor *γ*. Indeed, the CO dominates in a large part of the parameter space with low values of *γ*, and conversely is less powerful when *γ* is high (see the Supplementary Fig. S2).

**Figure 2:**
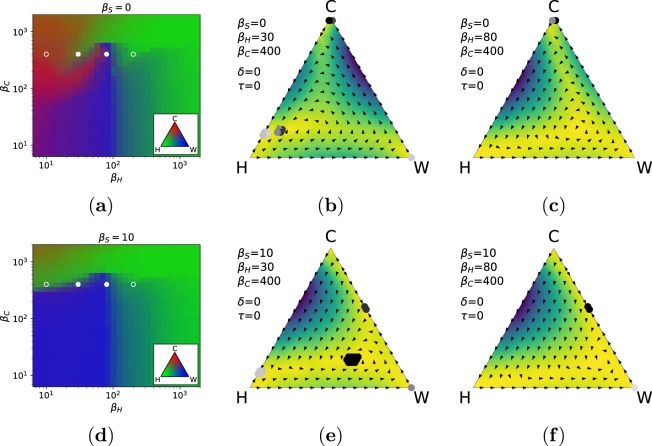
Effect of punishment from honest individuals (*β*_*H*_) and criminal organisation (*β*_*C*_) when the state is absent (*β*_*S*_ = 0, top row) or when it is present but weak (*β*_*S*_ = 10, bottom row). (**a**,**d**) Stationary distribution for different values of *β*_*H*_ and *β*_*C*_. Circles represent relevant configurations shown in the side panels, or in Supplementary Fig. S3. (**b**,**c**,**e**,**f**) Dynamics of the model under specific parametrisations. See Fig. 1 for additional details. Parameters of the model: *γ* = 0.5, *N* = 10, *c*_*W*_ = *c*_*C*_ = *r*_*W*_ = *r*_*C*_ = 1, *Z* = 50.

Slightly different dynamics are observable when punishment comes also from the civil society (i.e., *β*_*H*_ > 0). When the state organisation is absent (*β*_*S*_ = 0, see Fig. 2a), crime control falls on the shoulder of honest individuals, and their effectiveness is proportional to the size of the honest population. Hence, larger punishment values are required to produce a similar effect as with the state organisation. Also in such conditions, criminals prevail for low values of punishment *β*_*H*_, and are then gradually replaced by wolves when the punishment increases. Punishment from criminals to lone wolves provides an advantage to the former as long as *β*_*H*_ remains sufficiently small (see Fig. 2b). However, instead of having a clear phase transition to the domination of wolves, now spirals in the population dynamics appear (see Fig. 2c). In the presence of many honest individuals, criminals are those that lose out first, as they are easily victim of investigations. When the fraction of criminals is low, they do not sufficiently contribute to control the wolves, which can therefore dominate. However, as soon as honest are not enough to be exploited and to keep criminals under control, the residual punishment from criminals leads again to the recovery of the CO. Figure 2c shows that these dynamics are captured by a slow repelling spiral for *β*_*S*_ = 0, eventually leading to the dominance of organised crime. Depending on the specific value of *β*_*H*_ and *β*_*C*_, the final state can change favouring the one or the other group (see also the Supplementary Fig. S3a,b). The combined action of punishment from the state organisation and from the civil society has a strong effect on the subsistence of the CO (see Fig. 2d, where *β*_*S*_ = 10). In this case, lone wolves dominate in a large region of the parameter space. We can observe again spiralling dynamics (see Fig. 2e), which however tend to converge to a mixed equilibrium with many wolves and few honests and criminals. With increasing punishment from the civil society, the equilibrium shifts in favour of wolves first, and honest individuals later (see Fig. 2f and the Supplementary Fig. S3). We have also observed that criminals can coexist in a stable way with both wolves and honest. However wolves and honests are not able to coexist and maintain an equilibrium: if both are present in the population, they also need criminals for a stable coexistence.

As already mentioned, the parameter *γ* influences the success of the CO over lone wolves: the lower the punishment towards co-offenders, the higher the power of the criminal organisation with respect to lone wolves (see Supplementary Fig. S2). The size of the groups *N* also significantly affects the population dynamics: the larger the groups, the larger the benefit for victimisers, especially for wolves as shown in the Supplementary Fig. S4. In order to obtain a better insight, one can deduce how much punishment is necessary for eradicating victimisers under the limit of *N* = *Z* and when only two types of individuals are in the population (note that most of the resting points are found on the borders of the simplexes, hence justifying this assumption). Comparing the payoffs ${\omega }_{k}^{A}+{\omega }_{k}^{I}$ from Eqs 3–8 of each two pair of types of actors (and assuming for simplicity that *r*_*C*_ = *r*_*W*_ = 1, *c*_*C*_ = *c*_*W*_ = *c*, and *δ* = *τ* = 0), we obtain that:

- If *x*_*C*_ = 0, honest individuals defeat wolves if *β*′ > *N*^2^.
- If *x*_*W*_ = 0, honest individuals overcome criminals if *β*′ > (*g* *p*_*C*_)^−1^.
- If *x*_*H*_ = 0, criminals defeat wolves if *β*_*C*_ > (*N*^2^*g* *p*_*C*_ − 1)*β*_*S*_.

Where *β*′ = (*β*_*S*_ + *β*_*H*_*x*_*H*_)*c*^−1^ and *g* = (1 − *γ*)*p*_1_ + *γp*_*C*_. In a population with only wolves and honest individuals, the punishment *β*′ required for the latter to overcome the former is proportional to *N*^2^, confirming that larger groups provide a benefit to wolves. If instead of wolves the victimisers are only criminals, that punishment depends on how trackable are criminals, as determined by the parameter *γ*: if from one criminal it is easy to catch the others (*γ* ≈ 1 → *g* ≈ *p*_*C*_), the punishment *β*′—required to observe honest individuals prevailing on criminals—decreases with the square of the fraction of criminals ($\beta ^{\prime}  > {p}_{C}^{-2}$), whereas in the opposite case (*γ* ≈ 0 → *g* ≈ *p*_1_) this punishment decreases with the fraction of criminals but increases with the size of the population ($\beta ^{\prime}  > N\,{p}_{C}^{-1}$). This illustrates why both victimisers increase their power in bigger groups, but wolves do that in a greater way. Finally, in the competition between wolves and criminals, the latter gets a disadvantage under strong punishment from the state organisation, as well as when *γ* is high: for *γ* ≈ 1, the punishment that criminals have to inflict to wolves is ${\beta }_{C} > ({N}_{C}^{2}-1){\beta }_{S}$, scaling quadratically with the size of the CO, so as to compensate the costs paid from the investigation stage.

To understand under what conditions the criminal strategy is viable, we tested different parametrisation for the system by varying the level of harm inflicted by criminals and wolves during the acting stage, and the relative benefit they obtain from it (see Supplementary Fig. S5). In all cases, the stationary distribution is similar to the main cases described above, although scaled in favour of the one or the other type of victimiser, as expected by the relative strength provided by different costs-to-benefit ratios. Overall, following a CO pays off both when the harm inflicted is higher, as well as when the resulting reward is bigger than those of wolves. This means that, under the considered conditions, the CO needs to get some advantage in terms of professionalisation of the criminal activities in order to be sustainable.

**Figure 3:**
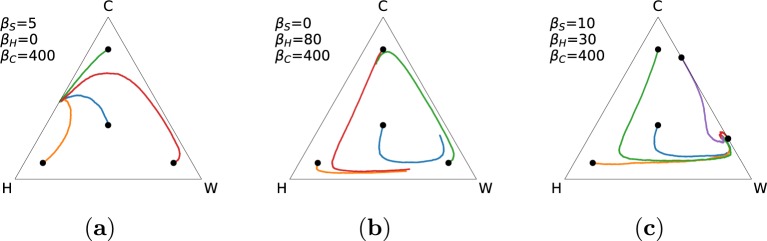
Dynamics resulting from Monte Carlo simulations for different values of *β*_*S*_, *β*_*H*_, and *β*_*C*_. Different colours show the dynamics from different initial conditions (marked here with a black filled circle) averaged over 500 realisations. Parameters of the model: *δ* = 0, *τ* = 0, *γ* = 0.5, *N* = 10, *c*_*W*_ = *c*_*C*_ = *r*_*W*_ = *r*_*C*_ = 1.

A set of Monte Carlo simulations were performed ratifying the results obtained using mean-field approximation. In Fig. 3, three different conditions are shown, respectively corresponding to Figs 1c and 2c,e. Each trajectory has been obtained by averaging over 500 independent runs, each run lasting 50000 iterations. At each iteration, *G* = 100 games have been performed to compute the average payoff of each individual within the population. The average trajectories shown in Fig. 3 closely correspond to the theoretical predictions, although the spiral center in Fig. 3c is shifted to the right.

### Terrorist networks

To model the terrorist scenario, we assume that criminals are terrorists that belong to an organised network, while wolves are terrorists that act on their own. We use the same names to refer to them in spite of the different roles they represent. With respect to the organised crime case, there are three important aspects that need to be taken into account. First, criminals organised in a TN have aligned interests with lone wolves. Both are willing to threaten and destabilise society, and are therefore not in competition. To model this aspect, we remove the direct punishment from criminals to wolves (*β*_*C*_ = 0). Second, the TN can directly benefit from criminal activities perpetrated by lone wolves, by gaining in power and in reputation at the expenses of the individuals that are directly involved in the terrorist act, who sometimes even sacrifice their lives for the cause. We model similar aspects of the terrorist interaction as a transfer of benefit from wolves to criminals by a fraction *τ* ∈ [0, 1], where a value of 1 implies a full transfer of benefit. Third, we consider the possibility that the TN actively promotes actions by lone wolves through their propaganda against the state. This is an effective strategy that can be modelled by linking the probability that lone wolves commit a terrorist act to the size of the TN: the larger the network, the stronger the propaganda, the more likely lone wolves act. We model this through the parameter *δ* linking the likelihood of wolves’ action to the fraction of criminals in the group.

**Figure 4:**
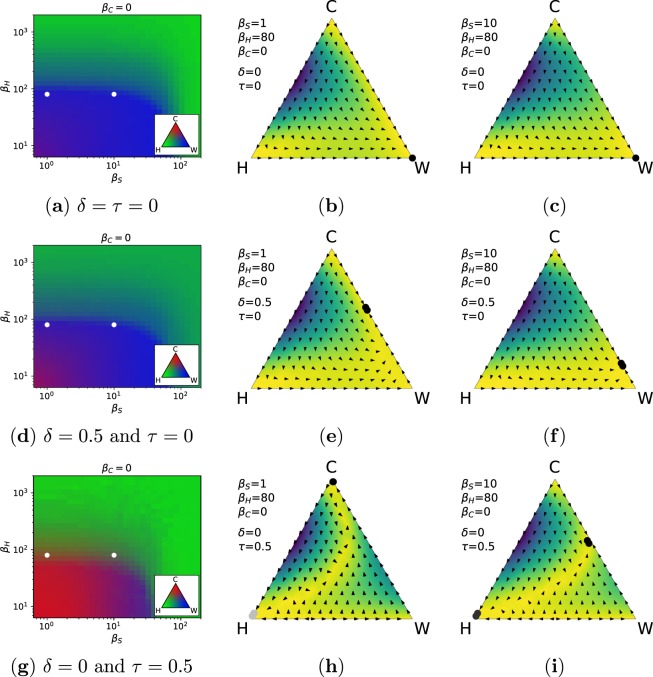
Effect of the propaganda (*δ*) and transfer of benefit (*τ*) in modelling the dynamics of TNs. We consider a baseline case in which neither of the proposed mechanisms are present (*δ* = 0 and *τ* = 0, first row), the case in which only propaganda is used (*δ* = 0.5 and *τ* = 0, second row), and the case in which only the transfer of benefit is considered (*δ* = 0 and *τ* = 0.5, third row). The combined effects of both mechanisms is shown in the Supplementary Fig. S6. (**a**,**d**,**g**) Each panel shows the stationary distribution of the different roles, similar to Fig. 1a, but for *β*_*C*_ = 0 and different values of *β*_*H*_ and *β*_*S*_. Filled circles represent relevant configurations displayed. (**b**,**c**,**e**,**f**,**h**,**i**) Dynamics of the model under relevant parametrisations.

The effect of the parameters *τ* and *δ* on the stationary distribution of roles within the population reveals that propaganda alone does not determine the dominance of the TN within the population (see Fig. 4). More specifically, we can observe that, when there is no propaganda and no transfer of benefit, the TN persists only for very low values of punishment *β*_*S*_ from the state organisation and *β*_*H*_ from the civil society (see Fig. 4a). Otherwise, wolves take over the entire population (see for instance the dynamics displayed in Fig. 4b,c), unless punishment is high enough (*β*_*S*_ > 100 or *β*_*H*_ > 1000).

## Conclusions

We have presented an evolutionary model that grasps interesting dynamics related to the proliferation of organised crime and terrorism in a population. The model accounts for offences perpetrated by criminals, individually or organised into criminal organisations or terrorist networks, at the expenses of other agents in the population. Different forms of punishment are considered to control the spread of crime into the modelled society, including social control by institutions and the civil society as well as punishment from organised criminals towards lone wolves.

A close look to the results presented in this study provides support to criminological theories about the development of organised crime and terrorist networks. Related to organised crime, our model predicts the establishment of a CO as an entity capable of providing protection to the population^41,42^, especially when they face a relatively weak state and when the civil society does not participate to punishment. The CO in this case takes the role of governance, controlling the criminal acts from wolves to impose their own rules. When instead honest citizens oppose themselves to the CO, an equilibrium is unlikely and cycles may be observed in which criminal activities grow and shrink in response to the reaction from the civil society. Similar complex patterns are found also in other modelling approaches to studying crime^21^, however in this case they are the result of dominance among different strategies that are made possible by frequency-dependent punishment, which introduces non-linearities that are relevant for the emergence of complex dynamical patterns.

Related to terror networks, we observed how the role of propaganda is central in promoting a stable equilibrium between the TN and the lone wolves, more than a possible transfer of benefit from wolves to the TN. Propaganda is recognised as a central aspect for terrorism, and is found in all its manifestations, especially recently with the strong exploitation of the Internet and of social media technologies^46^. The latter provide a rather cheap mean of publicising terror strategies, although their effect in effectively promoting radicalisation and terrorist acts still needs to be ascertained^47^. The model predicts that, when propaganda is successful, the TN can remain relatively small in size, but the terror goal is anyway reached thanks to the action of lone wolves. There is instead less evidence of a direct transfer of benefit from wolves to the TN other than a return in reputation and strength of the TN that claims responsibility on the attack, which however can be framed in a sort of competition between the terrorist organisation and the lone wolves^48^. Ultimately, criminology research confirms that any TN needs lone wolves either as autonomous cells that can perpetrate terror acts on their own, or as possible recruits to increase their dimension and power^43,44^. The proposed model can help in grounding different conceptual frameworks into tangible social dynamics.

Overall, despite being conceptually very simple, the proposed model grasps interesting patterns related to the development of CO and TN when embedded in a society in which also non-organised criminal activities are present. Real-world scenarios are clearly more complex than what pictured here, as they can involve both competition and collaboration between different criminal organisations, which can behave in a range of different ways against non-organised criminals, at times punishing, ignoring or promoting their activities. For instance, in our model, we do not consider the possibility of retaliation, which has been found to be relevant in other studies on crime dynamics^9,19^ and can be related to theoretical studies of antisocial punishment^49–52^. Nevertheless, the dynamics we observe are already rich enough to provide useful accounts on the underlying CO/TN dynamics, which could be matched with real-world instances. In this respect, an interesting possibility to study the dynamics of organised crime with respect to non-organised criminals is looking at the transplantation of a CO, whereby new territories not occupied by other COs are colonised by branches of organisations elsewhere very powerful^53^. Similar conditions offer an interesting opportunity to contrast the prediction from the proposed model to real-world observations, pointing to the need of collecting precise data about the spread of COs in correspondence to transplantation attempts.

The complex dynamical patterns that emerge from the study point to the need to take into account the social context in which certain criminal behaviour are observed. Indeed, more than individual predisposition to criminal activities, the theory of *social opportunity structure*^54^ postulates that involvement into criminal activities is strongly determined by social contacts and contingent opportunities that can become available at any point in the life of an individual. The evolutionary perspective that we take in this paper follows from the explanations provided by the social opportunity structure, as the mutation towards criminal activities is determined by the opportunities that are given by higher potential payoffs. To further build on this theory, models can be developed in which social ties are explicitly taken into account, for instance by having agents interact on heterogeneous networks, possibly changing over time to adapt to social contingencies, hence strengthening or loosing ties^32^. Interesting perspectives can be given by studying the evolutionary dynamics on multilayer networks^55,56^, which can grasp the existence of different types of relations between agents (family ties, friendship, work ties and so forth). In this way, it could be possible to include more detailed mechanisms for recruitment and radicalisation of individuals, further testing theories related to the social opportunity structure that accompanies the evolution of organised crime.

## Supplementary information

Supplementary Information
