# Supplementary material for: Evolutionary dynamics of organised crime and terrorist networks
Source: Sci Rep. 2019 Jul 5;9:9727. doi: 10.1038/s41598-019-46141-8 (PMC6611905; doi:10.1038/s41598-019-46141-8)
Supplement: Supplementary file 1 — Supplementary Information [file 41598_2019_46141_MOESM1_ESM.pdf]

Supplementary Information
Evolutionary dynamics of organised crime and terrorist networks

Luis Alberto Martinez-Vaquero*[1,2], Valerio Dolci[3,4] and Vito Trianni†[1]

[1]Institute of Cognitive Sciences and Technologies, National Research Council of Italy, via San Martino della Battaglia 44, 00185 Rome, Italy
[2]*Current address:* Lab of Socioecology and Social Evolution, Department of Biology, KU Leuven, Naamsestraat 59, 3000 Leuven, Belgium
[3]INFN Roma1, Rome, Italy
[4]Physics Department, Sapienza University of Rome, Italy

*l.martinez.vaquero@gmail.com
†vito.trianni@istc.cnr.it

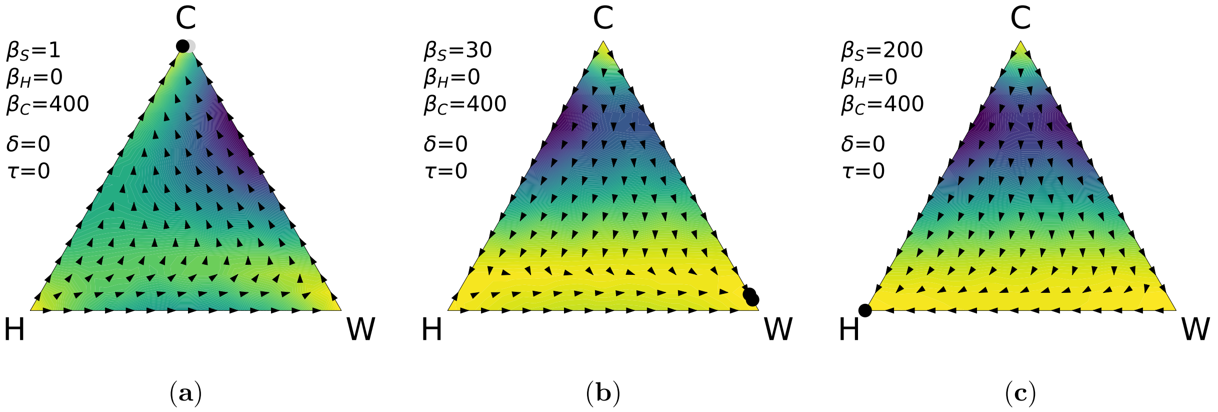

Supplementary Figure S1: Dynamics of the system under a strong punishment from criminals ($\beta_C = 400$) and varying punishment from the state organisation (β_S). For further details, refer to Figure 1 in the main text. Parameters of the model: $\gamma = 0.5$, $N = 10$, $c_W = c_C = r_W = r_C = 1$, $Z = 50$.

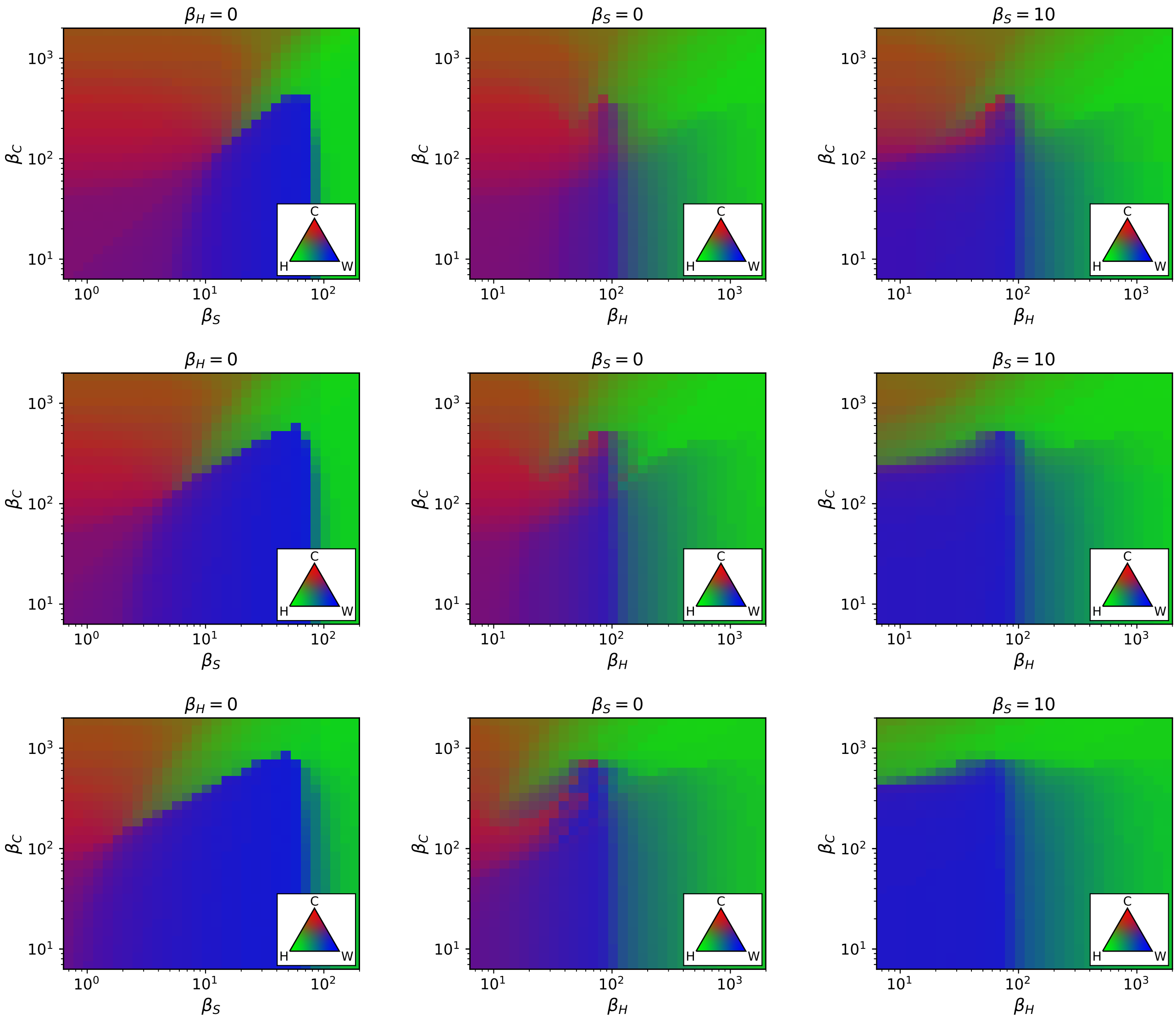

Supplementary Figure S2: Effect of the punishment inflicted to criminals other than the investigated one via the parameter γ. The higher the value, the more criminals are punished, providing an advantage to lone wolves. Three values of γ are tested here: $\gamma = 0$ (first row), $\gamma = 0.25$ (second row), and $\gamma = 1$ (third row). The stationary distribution is shown for different parameterisations, corresponding to Figure 1**a** (first column), Figure 2**a** (second column) and Figure 2**d** (third column). For further details, see Figure 1.

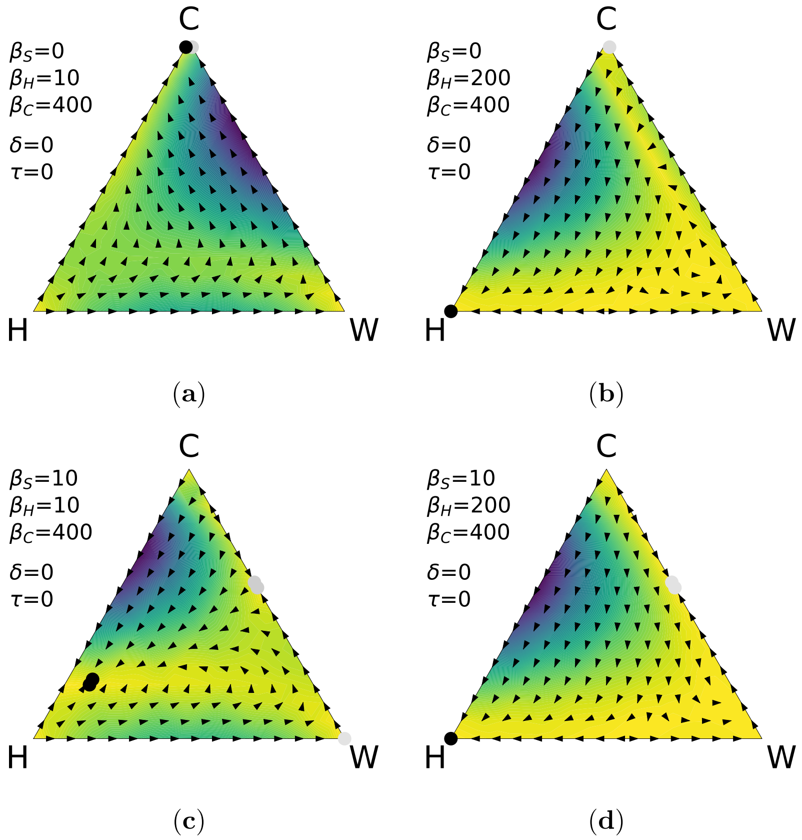

Supplementary Figure S3: Dynamics for relevant configurations as displayed in Figure 2**a** (first row) and Figure 2**b** (second row). For further details, see Figure 1.

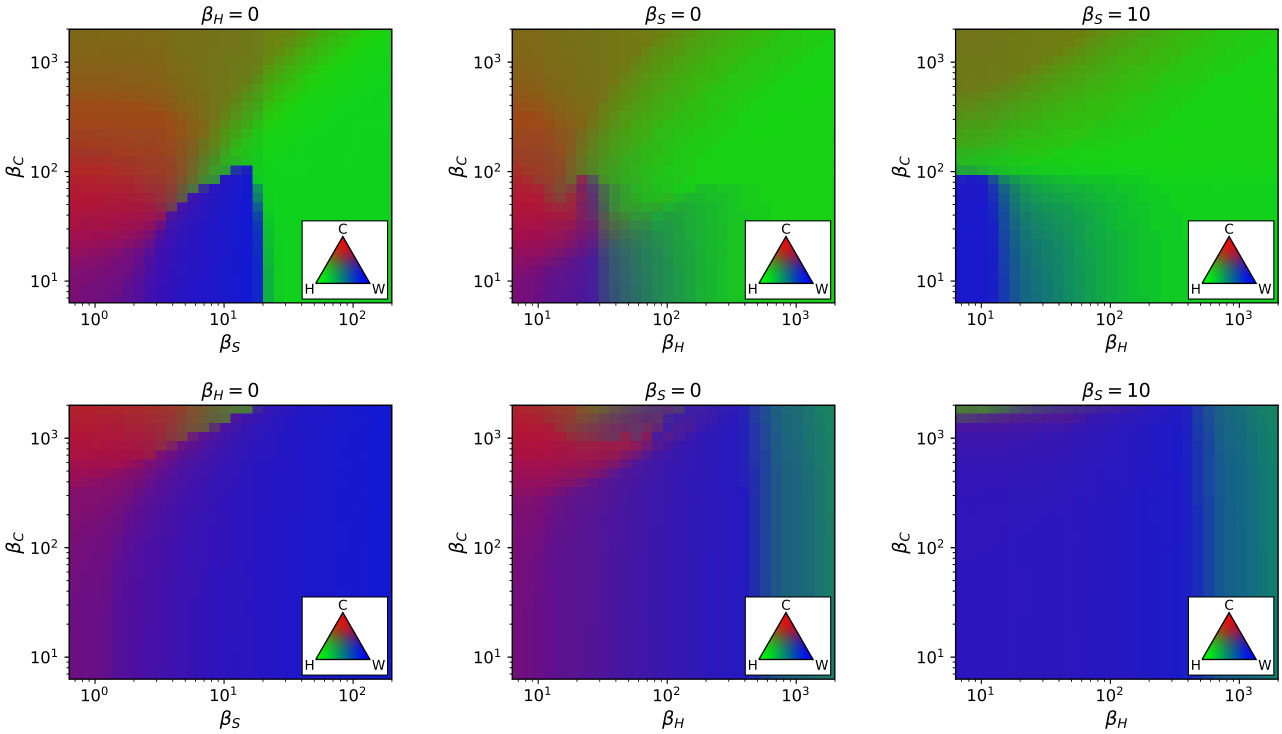

Supplementary Figure S4: Effect of the group size N. We vary the group size considering both smaller ($N = 5$, first row) and larger groups ($N = 25$, second row). The stationary distribution is shown for different parameterisations, corresponding to Figure 1**a** (first column), Figure 2**a** (second column) and Figure 2**d** (third column). For further details, see Figure 1.

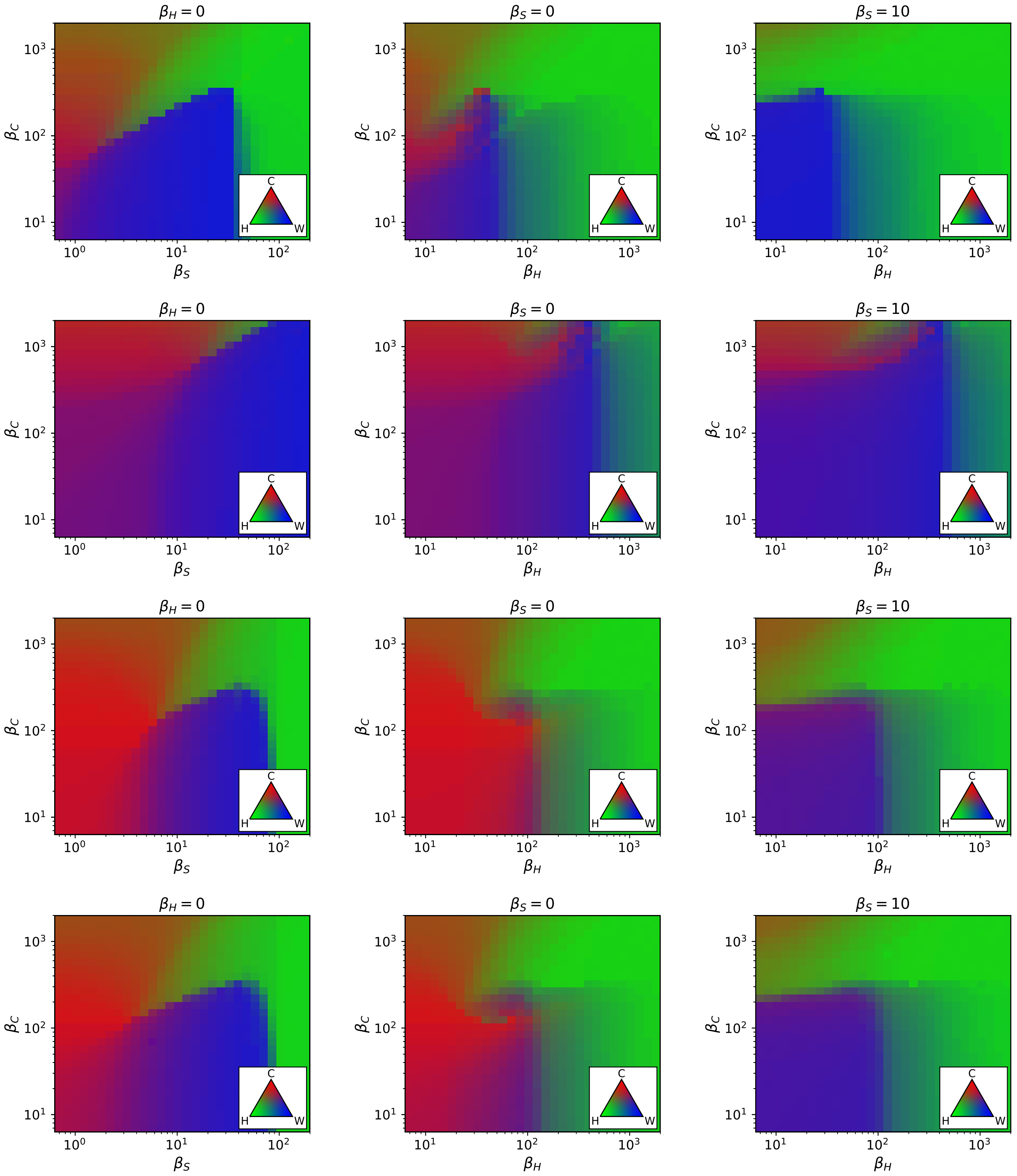

Supplementary Figure S5: Effect of the harm imposed by the victimisers and the benefit obtained out of it. We investigate the following conditions: $c_W = c_C = 0.5$ (first row), $c_W = c_C = 5$ (second row), $c_W = 1$ and $c_C = 2$ (third row), and $r_W = 1$ and $r_C = 2$ (fourth row). The stationary distribution is shown for different parameterisations, corresponding to Figure 1**a** (first column), Figure 2**a** (second column) and Figure 2**d** (third column). For further details, see Figure 1.

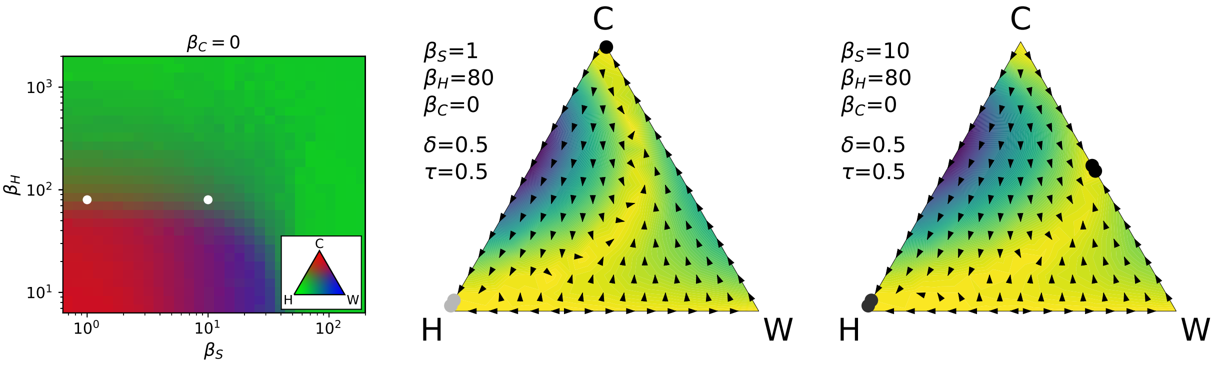

(**a**) $\delta = 0$ and $\tau = 0.5$ (**b**) (**c**)

Supplementary Figure S6: Combined effect of propaganda ($\delta = 0.5$) and transfer of benefit ($\tau = 0.5$) in modelling TNs. (**a**) Stationary distribution of the different roles, similar to Figure 1**a**, but for $\beta_C = 0$ and different values of β_H and β_S. (**b-c**) Dynamics for representative parameter configurations. See Figure 1 for further details.
